# Alterations of the Fecal Microbiota in Chinese Patients With Multiple Sclerosis

**DOI:** 10.3389/fimmu.2020.590783

**Published:** 2020-12-16

**Authors:** Zongxin Ling, Yiwen Cheng, Xiumei Yan, Li Shao, Xia Liu, Dajin Zhou, Lijuan Zhang, Kunqiang Yu, Longyou Zhao

**Affiliations:** ^1^ Collaborative Innovation Center for Diagnosis and Treatment of Infectious Diseases, State Key Laboratory for Diagnosis and Treatment of Infectious Diseases, National Clinical Research Center for Infectious Diseases, the First Affiliated Hospital, School of Medicine, Zhejiang University, Hangzhou, China; ^2^ Department of Laboratory Medicine, Lishui Second People’s Hospital, Lishui, China; ^3^ Hangzhou Normal University, Hangzhou, China; ^4^ Institute of Translational Medicine, The Affiliated Hospital of Hangzhou Normal University, Hangzhou, China; ^5^ Department of Intensive Care Unit, the First Affiliated Hospital, School of Medicine, Zhejiang University, Hangzhou, China

**Keywords:** multiple sclerosis, *Faecalibacterium*, IL-17, fecal microbiota, butyrate

## Abstract

Mounting evidence indicates that alterations in the intestinal microbiota may be associated with neurological disorders such as multiple sclerosis (MS). MS is a putative autoimmune disease of the central nervous system. However, it has not been determined whether the intestinal microbiota and host immune status are altered in Chinese patients with stable MS. In our study, 22 Chinese patients with stable MS and 33 healthy controls were enrolled for fecal microbiota analysis and host immunity evaluation. The microbial diversity and composition, bacterial co-occurrence correlations, predictive functional profiles, and microbiota-cytokine correlations between the two groups were compared. We observed that while the overall structure of the fecal microbiota did not change significantly, the abundances of several key functional bacteria, primarily *Faecalibacterium*, decreased remarkably. *Faecalibacterium* and *Granulicatella* could be used to distinguish between patients with MS and healthy controls with an area under the curve of 0.832. PiCRUSt analysis revealed that genes associated with fructose, mannose, and fatty acid metabolism were significantly enriched in the MS microbiota. In addition, we also observed that the levels of several pro- and anti-inflammatory cytokines and chemokines, such as IL-1ra, IL-8, IL-17, and TNF-α changed observably, and the abundances of key functional bacteria like butyrate producers correlated with the changes in the cytokine levels. Our present study indicated that altered composition of the fecal microbiota might play vital roles in the etiopathogenesis of MS by regulating host immunity, which suggests that microbiota-targeting patient-tailored early intervention techniques might serve as novel therapeutic approaches for MS.

## Introduction

Multiple sclerosis (MS) is a chronic inflammatory demyelinating disease of the central nervous system with an autoimmune mechanism of development, which is one of the leading causes of disability in young adults. MS affects approximately 2.3 million people worldwide, with a prevalence of 50–300/100,000 (1). The prevalence of MS has increased substantially in many regions since 1990, including China (2). Thus far, there are no comprehensive studies available on the national prevalence of MS. However, several regional studies have investigated the prevalence of MS in China and have indicated increasing prevalence over time (3–5). A study conducted by Cheng et al. revealed that the crude MS prevalence rate was 1.39 per 100,000 individuals in Shanghai in 2004 to 2005 and the female-to-male ratio was 1.8 (6). Another study estimated the prevalence of MS to be 3.7 and 6.7 cases per 100,000 individuals among males and females, respectively, based on hospital data for 2013 in Shandong Province (7). To date, more than 100,000 validated cases of MS have been diagnosed in China (2). Recently, there has been significant progress in the understanding of genetic and environmental factors underlying the condition. Compared to genetic factors, environmental factors, such as infection with Epstein-Barr virus, cigarette smoking, and low vitamin D levels owing to insufficient exposure to sunlight, play a greater role in susceptibility to the condition (8). However, the underlying cause of this disease remains elusive.

In humans, a complex cross-talk is required between the gut microbiota and the host immune system to maintain host homeostasis. The gut microbiota can influence the development of host immunity, and consequently, the immune system regulates the microbiota through gut barrier maintenance and immune exclusion. Mounting evidence suggests that the gut microbiota plays vital roles in various autoimmune disorders, including MS, which can contribute significantly to both susceptibility and protection (9–11). Studies using the experimental autoimmune encephalomyelitis model, which is most commonly used as an animal model of MS, have successfully confirmed that alterations in the gut microbiota are a potential risk factor for autoimmune diseases such as MS. Yokote et al. reported that the disruption of gut microbiota upon the administration of oral antibiotics reduces the severity of conventional experimental autoimmune encephalomyelitis (12). Moreover, using a relapsing-remitting mouse model of spontaneously developing experimental autoimmune encephalomyelitis, Berer et al. demonstrated that transgenic SJL/J mice raised in germ-free conditions are protected from the disease, while re-colonization with indigenous bacteria in the gut restored its susceptibility (13). These studies using animal models indicate that dysbiosis of the gut microbiota may play a central role in the development of MS.

Recently, several groups have profiled the fecal microbiota of patients with MS from western developed countries and have shown that patients with MS exhibit gut microbial dysbiosis with both depletion and enrichment of certain bacteria compared to healthy subjects. Jangi et al. reported an increased abundance of *Methanobrevibacter* and *Akkermansia* and decreased abundance of *Prevotella* in patients with MS. After successful treatment by disease-modifying therapy, the abundance of *Prevotella* increased significantly (10). Other studies also showed that the abundance of genus *Prevotella* decreases in patients with MS (9, 11). *Prevotella copri* has previously been found to be enriched in patients with new-onset rheumatoid arthritis, which is also an autoimmune disease (14). Therefore, the reduced abundance of *Prevotella* observed across multiple MS microbiome studies conducted in different geographical locations suggests that this bacterium might play an important role in MS. Miyake et al. (11) and Cantarel et al. (15) also observed that the abundance of *Fecalibacterium* was low in patients with MS compared to that in healthy controls. *Faecalibacterium prausnitzii* is an anti-inflammatory commensal bacterium identified in the gut microbiota analysis of patients with Crohn’s disease (16). In addition, Jangi et al. also observed alterations in the human gut microbiome in MS that correlate with changes in the host immune transcriptome and treatment. The gut microbiota can modulate host immune gene expression by establishing direct contact with cell wall components or by secreting factors such as short-chain fatty acids (SCFAs) (17, 18). Collectively, altered gut microbiota is considered to be related to MS pathogenesis and may serve as an important consideration in novel preventive or therapeutic strategies for MS in future.

To date, there have been no studies on the examination of gut microbiota in the Chinese MS-affected population. The environmental conditions, economic status, ethnic background, lifestyles, and long-term diets in China are distinct from those in western developed countries, which would affect the overall structure and composition of the gut microbiota and its roles and mechanisms in the pathogenesis of MS. In our study, we compared fecal microbiota specimens from patients with stable MS to those from healthy controls using high-throughput sequencing technique and attempted to explore the potential key functional fecal microbiota that were associated with MS in the patients studied. We observed that the abundance of several predominant genus-level taxa known to include butyrate producers decreased in the MS cohort, which indicated that altered composition of the gut bacteria might participate actively in the development of MS. Our study sheds light on the roles and mechanisms underlying the function of gut microbiota in MS pathogenesis, which will help identify novel microbiota-targeted biomarkers for noninvasive diagnosis and MS treatment.

## Materials and Methods

### Subjects’ Enrollment

The protocols for the present study were reviewed and approved by the Ethics Committee of Lishui Second People’s Hospital (Zhejiang, China). Informed written consent was obtained from each participant before enrollment. Twenty-two patients with MS, who were diagnosed based on the 2005 McDonald criteria (19), were recruited from Zhejiang province (China) from February 2019 to July 2019, and thirty-three age- and sex-matched healthy subjects were recruited as controls (**Table 1**). The cases were stable, and not of new onset or active relapse, and had not been treated with steroids, beta-interferon/glatiramer acetate, or other immunosuppressive medications in the preceding 3 months. The following exclusion criteria were established: age < 20 years; body mass index (weight in kilograms divided by height in meters squared) > 30; pregnancy; hypertension; diabetes mellitus; use of antibiotics, probiotics, prebiotics, or synbiotics in the previous month; known active infections such as bacterial, fungal, chlamydial, or viral infections; and other diseases such as irritable bowel syndrome, inflammatory bowel disease, or other autoimmune diseases.

**Table 1:** Summary of the study subjects’ characteristics.

Characteristics	Control (n = 33)	Patients (n = 22)
Age (means ± SD)	34.5 ± 8.2	35.0 ± 7.1
Gender(Female/male)	21/12	14/8
BMI(means ± SD)	23.85 ± 3.56	24.18 ± 3.24
Complications, no		
Hypertension	0	0
Diabetes mellitus	0	0
Hyperlipidemia	0	1
Irritable bowel syndrome	0	0
Autoimmune liver disease	0	0
Active infections	0	0
Antibiotics use within 1 month, no	0	0
Yogurt use within 1 month, no	0	0
Immunosuppressive medications within 3 months	0	0

BMI, body mass index; SD, standard deviation.

### Fecal Sample Collection and DNA Extraction

Approximately 2 g of a fresh fecal sample was collected in a sterile plastic cup, and stored at −80°C after preparation within 15 min until use. Bacterial genomic DNA was extracted from 300 mg of homogenized feces using a QIAamp^®^ DNA Stool Mini Kit (QIAGEN, Hilden, Germany) according to the manufacturer’s instructions, with additional glass-bead beating steps on a Mini-beadbeater (FastPrep; Thermo Electron Corporation, Boston, MA, USA). The amount of DNA was determined using a NanoDrop ND-1000 spectrophotometer (Thermo Electron Corporation); the integrity and size were checked by 1.0% agarose gel electrophoresis containing 0.5 mg/ml ethidium bromide. All DNA was stored at −20°C before further analysis.

### Amplicon Library Construction and Sequencing

Amplicon libraries were constructed with Illumina sequencing-compatible and barcode-indexed bacterial PCR primers 319F/806R, which target the V3–V4 regions of 16S rRNA gene (20). All PCR reactions were performed with KAPA HiFi HotStart ReadyMix using the manufacturer’s protocol (KAPA Biosystems) and approximately 50 ng of extracted DNA per reaction. Thermocycling conditions were set at 95°C for 1 min, 55°C for 1 min, then 72°C for 1 min for 30 cycles, followed by a final extension at 72°C for 5 min. All PCR reactions were performed in 50 μl triplicates and combined after PCR. The amplicon library was prepared using a TruSeq™ DNA sample preparation kit (Illumina Inc, San Diego, CA, USA). Prior to sequencing, the PCR products were extracted with the MiniElute^®^ Gel Extraction Kit (QIAGEN) and quantified on a NanoDrop ND-1000 spectrophotometer (Thermo Electron Corporation) and Qubit 2.0 Fluorometer (Invitrogen). The purified amplicons were then pooled in equimolar concentrations and the final concentration of the library was determined by Qubit (Invitrogen). Negative DNA extraction controls (lysis buffer and kit reagents only) were amplified and sequenced as contamination controls. Sequencing was performed on a MiSeq instrument (Illumina) using a 300 × 2 V3 kit together with PhiX Control V3 (Illumina) (21, 22).

### Bioinformatic Analysis

The 16S rRNA gene sequence data set generated from the MiSeq run were first merged and demultiplexed into per samples using the QIIME version 1.9.0 with default parameters (23). Chimera sequences were detected and removed using the USEARCH software based on the UCHIME algorithm (24). Open-reference operational taxonomic unit (OTU) pick was then performed with USEARCH V7 referenced against Greengenes database version 13.8 at 97% sequence similarity (25, 26). OTUs with a number of sequences <0.005% of the total number of sequences were discarded as recommended (27). The result was an OTU table, which was used for subsequent downstream analysis.

For taxonomic assignment, the most abundant sequences were chosen as the representative sequences of corresponding OTUs. Taxonomic assignment of individual datasets were classified against the Greengenes database version 13.8 using both RDP classifier and UCLUST version 1.2.22 methods implemented in QIIME (26, 28). Any sequences that were identified as members of Eukarya, Archaea, Mitochondria, Chloroplasts, and Cyanobacteria lineages, were removed. Alpha diversity was calculated with QIIME software with Python scripts base on the sequence similarity at 97% level, including index of observed species, abundance-based coverage estimator (ACE), Chao1 estimator, Shannon, Simpson, Evenness, and PD whole tree. Sequence coverage was assessed in mothur by rarefaction curves and Good’s coverage (29, 30). Beta diversity was measured by jaccard, bray-curtis, unweighted UniFrac, and weighted UniFrac distance calculated with 10 times of subsampling by QIIME. These distances were visualized by principal coordinate analysis (PCoA) (31). Hierarchical clustering was performed and heatmap was generated using a Spearman’s rank correlation coefficient as a distance measure and a customized script developed in the R statistical package. The output file was further analyzed using Statistical Analysis of Metagenomic Profiles software package (STAMP) version 2.1.3 (32).

For the predictive functional analyses, PiCRUSt software package version 1.0.0 was used to identify predicted gene families and associated pathways from inferred metagenomes of taxa of interest identified from the compositional analyses, which was based on the fact that phylogeny and function are closely linked (33). Predicted functional genes were categorized into Clusters of Orthologous Groups (COG) and into Kyoto Encyclopedia of Genes and Genome (KEGG) orthology (KO), and compared across patient groups using STAMP. Pathways and enzymes were assigned using KEGG database options built into the pipeline. The pathways that were nonprokaryotic, had fewer than two sequences in each cohort, or had a difference in mean proportions less than 0.1% were excluded from analysis. The characterization of microorganismal features differentiating the gastric microbiota was performed using the linear discriminant analysis (LDA) effect size (LEfSe) method (http://huttenhower.sph.harvard.edu/lefse/) for biomarker discovery, which emphasizes both statistical significance and biological relevance (34). With a normalized relative abundance matrix, LEfSe uses the Kruskal-Wallis rank sum test to detect features with significantly different abundances between assigned taxa and performs LDA to estimate the effect size of each feature. A significant alpha at 0.05 and an effect size threshold of 2 were used for all biomarkers discussed in this study.

Correlation analysis was performed using sparse compositional correlation (SparCC) algorithm on the complete OTU table collapsed to the genus level, which was introduced by Friedman and Alm and was known for its robustness to the compositional effects that are influenced by the diversity and sparsity of correlation in human microbiome data sets (35). SparCC was employed to represent co-abundance and co-exclusion networks between OTUs. For SparCC, 1000 bootstrap replicates were used to calculate significance values, and considered correlation coefficients greater or less than 0.2 and −0.2, respectively, and p-values <0.05. This set of iterative procedures were applied separately to normal, peritumor and tumor data sets to infer the basis correlation values within and/or between paired sampling sites. Visualization of the network was achieved using Cytoscape version 3.4.1.

### Systemic Inflammatory Cytokines Analysis

Serum samples from these participants were obtained using their fasting blood in the early morning. Using a 27-plex magnetic bead based immunoassay kit (Bio-Rad, CA, USA), the following cytokines were quantified: interleukin-1β (IL-1β), IL-1 receptor antagonist (IL-1ra), IL-2, IL-4, IL-5, IL-6, IL-7, IL-8, IL-9, IL-10, IL-12(p70), IL-13, IL-15, IL-17, Eotaxin, Fibroblast growth factor-basic (FGF-basic), granulocyte colony-stimulating factor (G-CSF), granulocyte-macrophages colony-stimulating factor (GM-CSF), interferon gamma (IFN-γ), interferon gamma-inducible protein 10 (IP-10), monocyte chemotactic protein-1 (MCP-1), macrophages inflammatory protein-1α (MIP-1α), platelet-derived growth factor (PDGF-bb), MIP-1β, regulated upon activation normal T-cell expressed and secreted (RANTES), tumor necrosis factor-alpha (TNF-α), and vascular endothelial growth factor (VEGF). The Bio-Plex 200 system was utilized for the analysis of Bio-Rad 27-plex human group I cytokines and the Bio-Plex assay (Bio-Rad) was performed according to the manufacturer’s directions. The results expressed as picogram per milliliter (pg/ml) using standard curves integrated into the assay and Bio-Plex Manager v6.0 software with reproducible intra- and inter-assay CV values of 5% to 8%.

### Statistical Analysis

For continuous variables, independent *t*-test, White’s nonparametric *t*-test, and Mann-Whitney *U*-test were applied. For categorical variables between groups, Pearson chi-square or Fisher’s exact test was used, depending on assumption validity. For correlation analyses, Spearman’s rank correlation test was used. Statistical analysis was performed using the SPSS V19.0 (SPSS Inc., Chicago, IL) and STAMP V2.1.3 (32). GraphPad Prism version 6.0 (San Diego, CA) was used for preparation of graphs. All tests of significance were two sided, and p < 0.05 or corrected p < 0.05 was considered statistically significant.

### Accession Number

The sequence data from this study are deposited in the GenBank Sequence Read Archive with the accession number SRP258890.

## Results

### Overall Bacterial Diversity in Fecal Microbiota in Patients With Stable Multiple Sclerosis

In our present study, the possible confounders of microbiota analyses, such as sex, age, and BMI between patients with stable MS and healthy controls did not differ significantly (p > 0.05). To investigate the alterations in fecal microbiota between the patients and healthy controls, we obtained 1,360,209 high-quality reads with an average of 24,731 reads per sample for the subsequent microbiota analysis. Good’s estimator of coverage was 99.33%, which indicated that the identified reads represented the majority of bacterial sequences (909 OTUs identified) present in the fecal microbiota. The diversity indices and richness indices were used to assess the overall differences in microbial community structure between patients with MS and healthy controls. We observed that the alpha-diversity indices, such as Shannon and Simpson indices, did not differ significantly between patients with MS and healthy controls (**Supplementary Figures 1A–C**), and ACE and Chao1 for the observed species in patients with MS were similar to those in healthy controls (**Supplementary Figures 1D, E**). Owing to significant inter-individual variations, the two groups could not be divided into different clusters using principal coordinate analyses based on Jaccard, Bray-Curtis, unweighted UniFrac, and weighted UniFrac algorithms (**Supplementary Figures 1F–I**). However, the Adonis test yielded p-values of 0.009 for Jaccard and 0.012 for Bray-Curtis analyses, which indicated significant microbial differences between the two groups. The Venn diagram showed that 728 OTUs with a total richness of 909 were shared between the two groups, with less unique OTUs in patients with MS (**Supplementary Figure 1J**). The rarefaction and rank abundance curves for patients with MS were also similar to those for healthy controls (**Supplementary Figures 1K, L**). The alpha - and beta-diversity analyses indicated that the overall diversity of MS-associated fecal microbiota remained unaltered; however, a lower number of OTUs and phylotypes were observed in patients with MS.

### Altered Composition of Fecal Microbiota in Patients With Multiple Sclerosis

**Figure 1:**
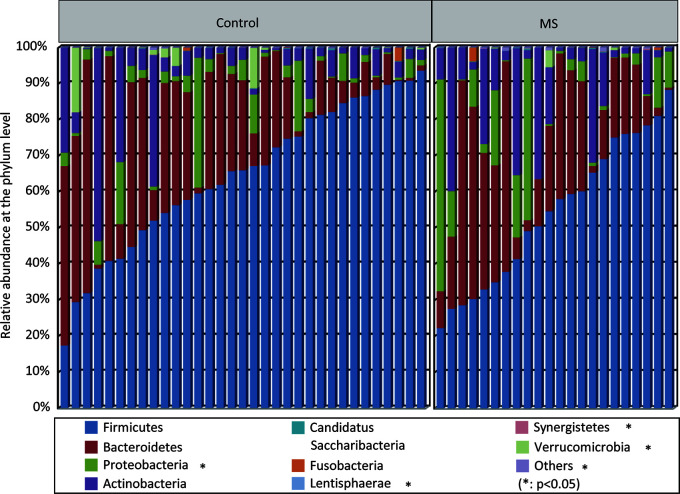
Variations in fecal microbial composition in Chinese patients with multiple sclerosis (MS). Relative proportions of bacterial phyla in patients with MS (n = 22) and in healthy controls (n = 33).

**Figure 2:**
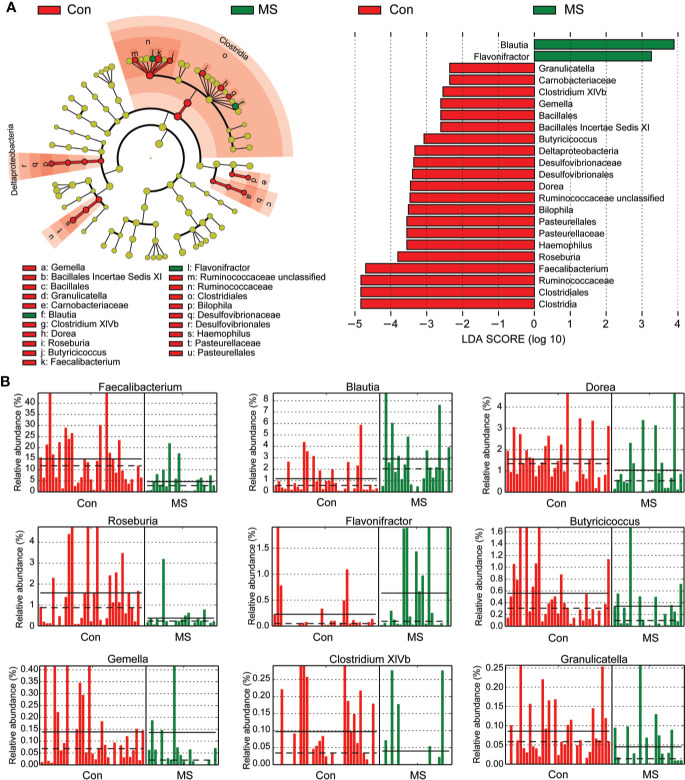
Different bacterial taxa between Chinese patients with multiple sclerosis (MS) and healthy controls. LEfSe identifies the taxa with the greatest differences in abundances between Chinese patients with MS and healthy controls. Only the taxa that meet a significant LDA threshold value of > 2 are shown **(A)**. Nine differentially abundant bacterial taxa were identified between the two groups **(B)**.

With the RDP classifier, sequences from the fecal microbiota could be classified among nine phyla; Firmicutes and Bacteroidetes were the predominant phyla in both groups. **Figure 1** shows that the abundances of Proteobacteria, Lentisphaerae, Synergistetes, and Verrucomicrobia, which were the non-dominant phyla, differed significantly between patients with MS and healthy controls (p < 0.05), while the ratio of Firmicutes/Bacteroidetes did not differ significantly between the two groups. In addition, 62 families and 149 genera were identified in the fecal microbiota specimens from both groups. **Supplementary Figure 2** shows a heatmap of bacterial families present in the microbiota in patients with MS and healthy controls, which represents the relative percentages of most families identified in each sample. Our data indicated that there were no significant differences in the heatmap between the two groups. Discriminant analyses using LEfSe showed that 21 bacterial phylotypes differed significantly between the two groups (LDA score > 2.0, p < 0.05, **Figure 2A**). Among the differentially functional bacterial taxa, only two taxa, including *Blautia* and *Flavonifractor* (belonging to Firmicutes) were enriched in patients with MS, while the abundances of other taxa, such as *Faecalibacterium*, *Roseburia*, *Haemophilus*, *Bilophila*, *Dorea*, *Butyricicoccus*, *Gemella*, *Clostridium* XIVb, and *Granulicatella*, decreased significantly in patients with MS (**Figure 2B**). Notably, *Faecalibacterium*, *Roseburia*, *Dorea*, *Butyricicoccus*, and *Clostridium* XIVb (all belonging to Firmicutes) can produce various SCFAs, such as butyrate. The present data indicates that the compositional abnormalities observed in the fecal microbiota in patients with MS are associated with a reduced capacity for producing SCFAs, especially butyrate, in the presence of different substrates, which indicates that functional dysbiosis becomes more obvious as MS progresses.

**Figure 3:**
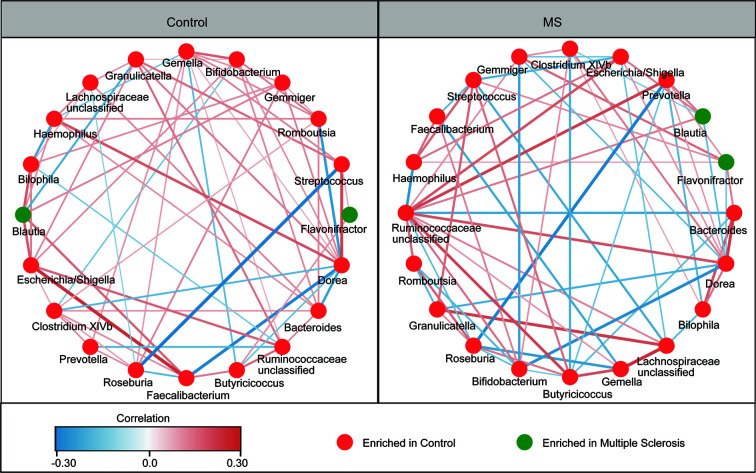
Correlation strengths of the abundant fecal microbiota in Chinese patients with multiple sclerosis (MS) and healthy controls. Correlation network of the abundant fecal microbiota in healthy controls and in patients with MS. The correlation coefficients were calculated using the Sparse Correlations for Compositional data (SparCC) algorithm. Cytoscape version 3.4.0 was used for network construction. The red and blue lines represent positive and negative correlations, respectively. The correlation networks were observed to become simpler.

The overall structure of the fecal microbiota results from dynamic interactions between members of the microbial community. A SparCC algorithm with false discovery rate adjustments was employed to generate correlation-based microbial interaction networks based on the relative abundance of OTUs between the two groups (**Figure 3**). We observed that the interaction networks in healthy controls are more complicated than those in MS patients. There were more positive correlations among the bacteria in healthy controls, while more negative correlations were observed in those in patients with MS.

**Figure 4:**
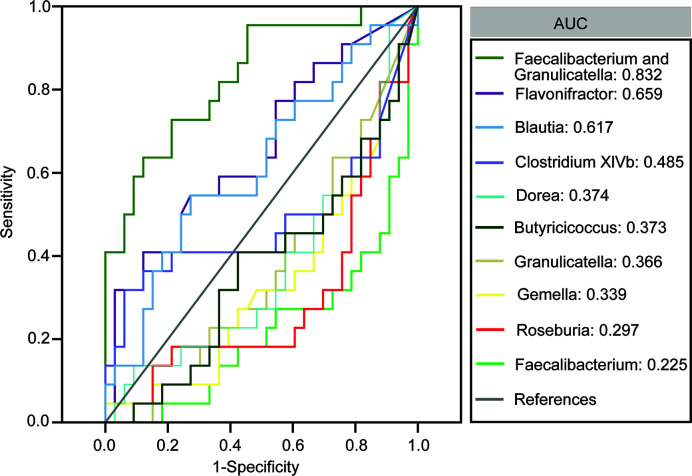
Receiver operating characteristic curves for differentially abundant genera such as *Flavonifractor, Blautia, Clostridium* XIVb, *Dorea, Butyricicoccus, Granulicatella, Gemella, Roseburia* and *Faecalibacterium, Streptococcus, Halomonas, Shewanella*, and *Ruminococcus* and *Dialister* that were used to distinguish between patients with multiple sclerosis and healthy controls.

We also assessed the potential of using fecal microbiota as biomarkers to distinguish between patients with MS and healthy controls. First, using a single differential bacteria as a predictor, we found that the area under the receiver operating characteristic curve ranged from 0.225 to 0.659 (**Figure 4**). Further, multivariable stepwise logistic regression analysis was performed using the MS-associated genera to identify taxa that helped create the most significant distinction between patients with MS and controls. We observed that the best and simplest combination was that of *Faecalibacterium* and *Granulicatella*, which could help distinguish between patients with MS and healthy controls with an area under the curve (AUC) of 0.832. However, the other combinations did not significantly improve the predictive performance (AUC ≤ 0.832).

### Functional Changes in Multiple Sclerosis-Associated Fecal Microbiota

**Figure 5:**
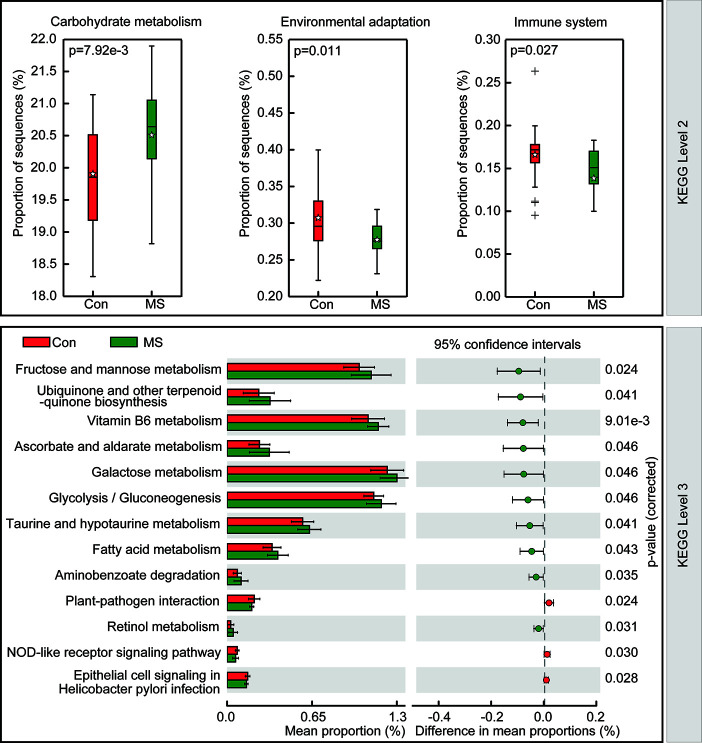
PiCRUSt-based study of fecal microbiome in Chinese patients with multiple sclerosis (MS) and healthy controls. The different bacterial functions were evaluated and compared between the two groups based on two-sided Welch’s *t*-test. Comparisons between the two groups for each KEGG functional category (levels 2 and 3) are indicated by the percentage. The Benjamini-Hochberg method was used for multiple testing correction based on the false discovery rate determined by STAMP.

To study the functional and metabolic changes in the microbial communities between MS and controls, we retrieved the metagenomes from the 16S rRNA data and analyzed the functional potential of the fecal microbiota using PiCRUSt based on closed-reference OTU picking. We compared 64 Kyoto Encyclopedia of Genes and Genome (KEGG) pathways at level 2 and identified three KEGG categories, carbohydrate metabolism, environmental adaptation, and immune system, with significantly different abundances between patients with MS and healthy controls (p < 0.05; **Figure 5**). Specifically, fructose and mannose metabolism, ubiquinone and other terpenoid-quinone biosynthesis, vitamin B6 metabolism, ascorbate and aldarate metabolism, galactose metabolism, glycolysis/gluconeogenesis, taurine and hypotaurine metabolism, fatty acid metabolism, aminobenzoate degradation, and retinol metabolism were significantly enriched at level 3 in the fecal microbiota of MS patients, while plant-pathogen interaction, epithelial cell signaling in *Helicobacter pylori* infection, and NOD-like receptor signaling pathway were significantly inhibited (p < 0.05). Collectively, these functional changes in the fecal microbiota may be associated with MS pathogenesis.

### Correlations Between Fecal Microbiota and Systemic Inflammation Among Patients With Multiple Sclerosis

**Figure 6:**
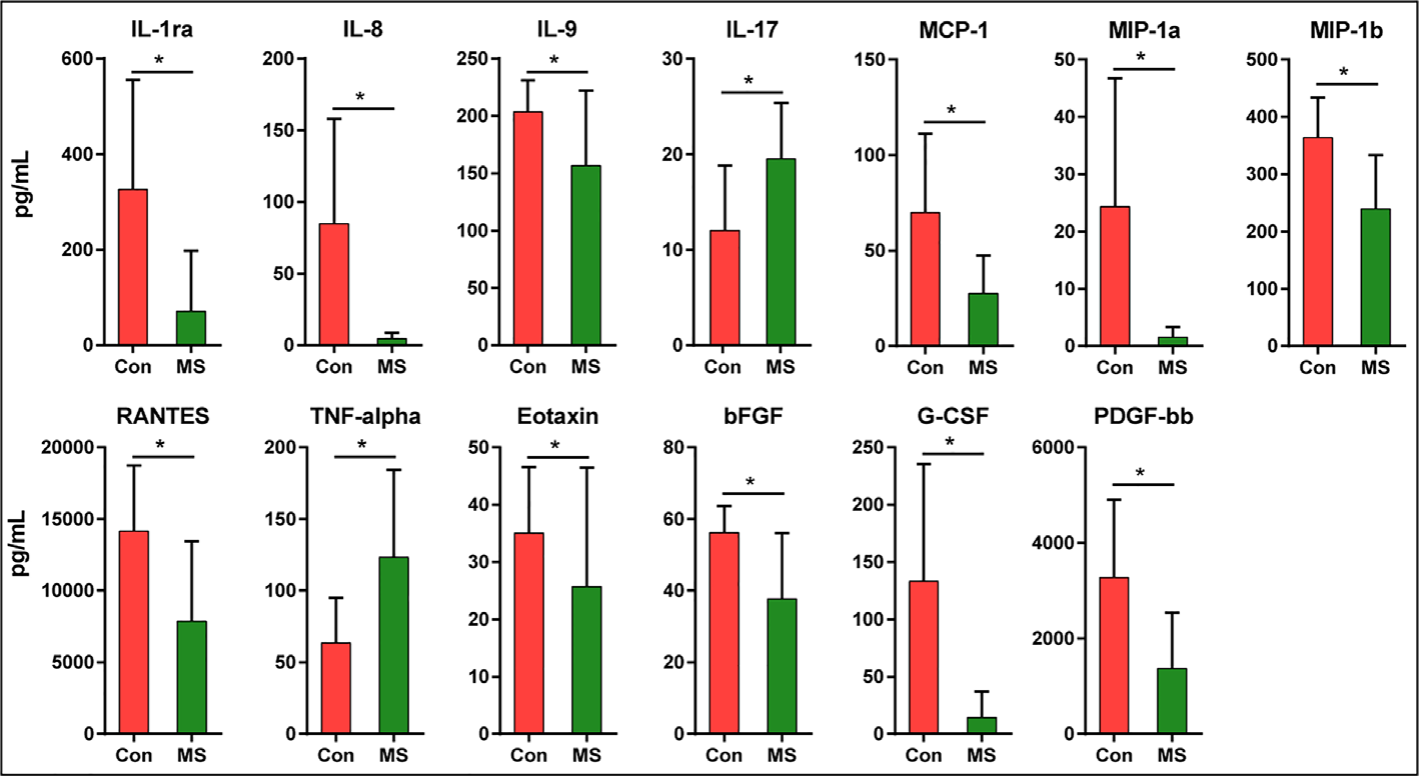
Mean (SEM) concentrations (pg/ml) of 27 pro- and anti-inflammatory cytokines and chemokines in Chinese patients with MS and in healthy controls determined using Bio-Plex immunoassays. The concentrations of TNF-α and IL-17 increased significantly in Chinese patients with MS, while those of IL-1ra, IL-8, IL-9, MCP-1, MIP-1a, MIP-1b, RANTES, Eotaxin, bFGF, G-CSF, and PDGF-bb decreased significantly. *p < 0.05.

**Figure 7:**
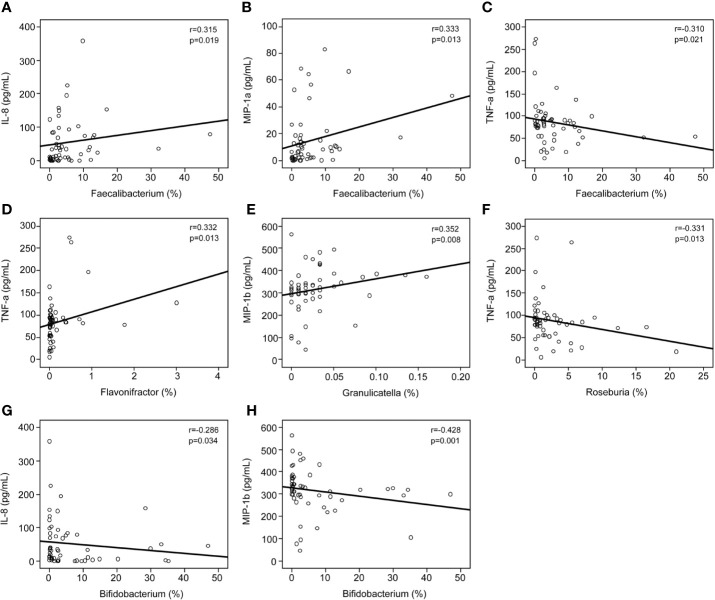
Correlations between pro- and anti-inflammatory cytokines and chemokines with altered concentrations and the relative abundance of the key functional genera. Correlation between the relative abundance of *Faecalibacterium* and the levels of IL-8 **(A)**, MIP-1a **(B)**, and TNF-α **(C)**; the relative abundance of *Flavonifractor* and the levels of TNF-α **(D)**; the relative abundance of *Granulicatella* and the levels of MIP-1b **(E)**; the relative abundance of *Roseburia* and the levels of TNF-α **(F)**; and the relative abundance of *Bifidobacterium* and the levels of IL-8 **(G)** and MIP-1b **(H)**. Spearman’s rank correlation (r) and probability (p) were determined to evaluate the statistical importance.

The findings of the Bio-Plex Pro™ human cytokine group I panel 27-plex analysis revealed that the concentrations of several cytokines changed significantly between healthy controls and patients with MS (**Figure 6**). In patients with MS, the levels of inflammatory cytokines, such as TNF-α and IL-17, increased significantly, while those of anti-inflammatory mediators IL-1ra and the chemokines IL-8, Eotaxin, RANTES, MIP-1a, MIP-1b, and MCP-1 decreased. In addition, the levels of growth factors such as bFGF, G-CSF, PDGF-bb, and pleiotropic cytokine IL-9 also decreased significantly in patients with MS. We also evaluated the correlations between the altered cytokine levels and the abundances of key functional bacteria among Chinese patients with MS (**Figure 7**). Notably, we observed that the abundance of butyrate-producing *Faecalibacterium* correlated positively with the levels of chemokines such as IL-8 (r = 0.315; p = 0.019) and MIP-1a (r = 0.333; p = 0.013), while it correlated negatively with those of inflammatory cytokines such as TNF-α (r = −0.310; p = 0.021). The abundance of *Flavonifractor*, which is enriched in MS, correlated positively with the levels of TNF-α (r = 0.332; p = 0.013), whereas the abundance of *Roseburia* correlated negatively with the levels of TNF-α (r = −0.331; p = 0.013). The abundance of *Granulicatella*, which is generally low in patients with MS, was also observed to correlate positively with the levels of MIP-1b (r = 0.352; p = 0.008). The abundance of the beneficial bacteria *Bifidobacterium* did not change significantly in Chinese patients with MS; however, it correlated negatively with the levels of IL-8 (r = −0.286; p = 0.034) and MIP-1b (r = −0.428; p=0.001). These correlations indicate that the key functional bacteria with altered abundances might regulate the inflammatory status in MS, at least partially, and consequently, may play a vital role in the progression, remission, and prognosis of MS.

## Discussion

Mounting evidence indicates that commensal microbiota that colonize the gastrointestinal tract play a vital role in regulating host immunity and maintaining host immune homeostasis. The host gut microbiota, which co-evolve tightly with the immune system, can modulate development and regulate innate and adaptive immunity functions, whereas dysbiosis of the gut microbiota facilitates abnormal immunological development (36). Research over the past decade has helped us appreciate the importance of commensal microbiota in the development of autoimmune diseases, such as inflammatory bowel disease, psoriasis/psoriatic arthritis, rheumatoid arthritis, systemic lupus erythematosus, celiac disease, and type 1 diabetes (37–43). As the most common autoimmune disease, MS targets the central nervous system and can lead to chronic disability, including cognition impairment, loss of motor control, and sensitivity (44, 45). MS is a multifactorial complex disease, and both genetic and environmental factors contribute to its pathogenesis (46). Similar to other gut-brain disorders, alterations in the relative proportions of gut microbiota in newly or active MS have been reported in several recent studies (9–11, 18, 46–49). Therefore, MS is also considered a type of gut-brain disorder.

In our previous studies, we reported that gut-brain disorders such as depression, Parkinson’s disease, Alzheimer’s disease, cerebral ischemia/reperfusion injury, and vascular dementia are associated with the dysbiosis of gut microbiota among Chinese individuals and animal models, which provides novel targets for the study of their pathogenesis and sheds light on novel intervention strategies (18, 50–57). The majority of studies on the “MS microbiome” conducted earlier primarily focused on Caucasian populations from western developed countries. However, there are several potential confounders, such as natural environmental conditions, economic status, ethnic background, lifestyles, and long-term diets that influence the bacterial composition in the gut; moreover, dietary patterns among Chinese populations, characterized by low calorie, low fat, and high dietary fiber, are different from those among Caucasian populations. Given that different populations have diverse gut microbiota, studies that target a specific population are necessary to decipher the mechanism underlying pathogenesis and identify therapeutic strategies more precisely. Therefore, the specific differences in gut microbiota that result from different environmental factors such as dietary habits may interfere with the development of MS, and we speculate that there may be specific changes in the gut microbiota in Chinese patients with stable MS. In the present study, we observed that the overall bacterial diversity in the fecal microbiota did not change significantly between the healthy cohort and MS patient cohort. The alpha-diversity indices, such as the Shannon and Simpson indices, did not undergo obvious changes, and the richness indices, such as observed OTUs, ACE, and Chao1, were also not altered significantly. In addition, the groups could not be divided into different clusters based on the beta-diversity indices. Consistent with the findings of a previous study by Jangi et al. (10), the present study showed that the overall bacterial diversity in the fecal microbiota was unaltered between healthy controls and Chinese patients with MS, which suggests that the overall structure of the fecal microbiota remained relatively stable among Chinese patients with MS.

Although the overall bacterial diversity of the fecal microbiota remained unaltered among Chinese patients with MS in this study, based on statistical analysis, compositional changes in the fecal microbiota were observed to be associated with MS development. At the phylum level, the abundances of several non-abundant phyla such as Proteobacteria, Lentisphaerae, Synergistetes, and Verrucomicrobia were observed to be altered, while the ratio of Firmicutes/Bacteroidetes, the two most prevalent phyla, did not change significantly. Lopez et al. demonstrated that a reduced Firmicutes/Bacteroidetes ratio was observed in patients with systemic lupus erythematosus (58), which indicates that gut dysbiosis is a major feature in this autoimmune disease. However, the composition of the fecal microbiota at the family and genus levels changed significantly in Chinese patients with MS. We also observed a more complex network of interactions among the differentially abundant bacteria in healthy controls than in patients with MS. According to our findings, the most important features of the specific “MS microbiota” among Chinese patients were characterized by a reduction in the abundances of bacteria belonging to *Faecalibacterium*, *Roseburia*, *Dorea*, *Butyricicoccus*, and *Clostridium* XIVb, which were potent producers of SCFAs, especially butyrate, and ferment indigestible carbohydrates to produce SCFAs. Although we did not measure the altered levels of SCFAs in the fecal samples directly, the altered profiles of the fecal microbiota provided some clues that fecal SCFAs might involve in the development of MS. In fact, the types and concentrations of SCFAs in the gut depend on the composition of the microbiota, the intestinal transit time, and the fiber content in the host diet. In addition to their role as local energy carriers for the gut microbiota and gut epithelial cells, SCFAs also play roles in a broad array of functions that influence gastrointestinal physiology, peripheral immunity, liver metabolism, and blood-brain barrier integrity, which could indirectly contribute to effects on the brain (59). SCFAs can promote health at a steady state by increasing intestinal epithelial cell integrity, pathogen-specific antibody responses, and the number of colonic Tregs, which can act as important signals that physically bridge the gap between the commensal microbiota and the mucosal immune system (60–64). Tregs play a critical role in immune homeostasis and in suppressing excessive immune responses elicited by the host. Kim et al. found that SCFAs are able to induce metabolic and epigenetic reprogramming linked to the suppression of inflammatory immune responses (65), which are essential for the development of autoimmune diseases. Mizuno et al. demonstrated that the oral administration of SCFAs ameliorates the severity of systemic autoimmune inflammatory conditions mediated by lymphocytes, such as experimental autoimmune encephalitis and collagen-induced arthritis. Amelioration of a disease is associated with a reduction in the number of Th1 cells and an increase in those of Treg cells (66). Tregs, the regulation of which is driven by the transcription factor Foxp3, are particularly important for limiting autoimmunity and chronic inflammation (67, 68). Therefore, alterations in the production of potent SCFAs and their metabolites by the fecal microbiota might contribute significantly to MS development, and could be used as a potential therapeutic target for MS in future studies.

In fact, gut dysbiosis and the consequent increase in gut permeability can lead to the disruption of immune homeostasis, following the development of gut inflammation, which is increasingly considered to be the ultimate source of systemic immune activation and Th17/Treg cell imbalance, and possibly of neurological disturbances as well. Consistent with the findings of a previous study, the relative abundance of *Faecalibacterium* (a member of *Clostridium* cluster IV) reduced significantly, which can convert acetate and lactate into butyrate (10). The most important properties of *Faecalibacterium* are reported to be their anti-inflammatory characteristics and the ability to attenuate inflammation in mouse models of colitis, which is mediated by the modulation of the mucosal T cell response (69). A previous study reported that the supernatant of *Faecalibacterium* culture, primarily the metabolite butyrate, can maintain Th17/Treg balance and ameliorate colorectal colitis by inhibiting histone deacetylase 1 (HDAC1) (70); this is of significance because Th17/Treg cell imbalance has been implicated in the pathogenesis of most common autoimmune diseases, including MS (71). Furusawa et al. reported that treatment of naïve T cells with butyrate, which is an HDAC inhibitor, enhanced the acetylation of Foxp3, and consequently promoted the differentiation of Treg cells (72). The abundance of *Clostridium* XIVb, which is another butyrate producer, was observed to decrease significantly in Chinese patients with MS. In our previous study, we observed a reduction in the relative abundance of *Clostridium* XIVb in patients with major depressive disorder, which correlated negatively with the serum BDNF levels (55). Inconsistent with our present findings, the abundance of *Clostridium* XIVb was observed to increase in various diseases such as juvenile idiopathic arthritis and Rett syndrome (73, 74). Similar to *Clostridium* cluster IV, *Clostridium* XIVb was the most effective in generating high levels of Treg cells in the CD4^+^ T cell population, which are essential for maintaining immune tolerance and abrogating chronic inflammatory or autoimmune diseases (75). In addition, our study also demonstrated the reduction in the abundance of *Roseburia*, a member of the *Clostridium* cluster XIVa, in Chinese patients with MS. Similar to *Faecalibacterium*, the butyrate producer, *Roseburia*, was also detected in patients with Crohn’s disease. Machiels et al. reported that the abundances of both *Faecalibacterium* and *Roseburia* correlate inversely with disease activity in ulcerative colitis (76). *Roseburia* has been shown to exert beneficial effects in colitis model mice and Caco-2 cells by enhancing the anti-inflammatory response, which could reduce the DAI score by enhancing colonic Treg cell differentiation and the levels of the anti-inflammatory cytokines TSLP, IL-10, and TGF-β in the intestinal mucosa of colitis model mice (77). Therefore, *Roseburia* also played a beneficial probiotic role in alleviating inflammation in autoimmune diseases. *Dorea*, which is also a member of the *Clostridium* cluster XIVa, has been considered to form a part of healthy gut microbiota. Chen et al. found that the relative abundance of *Dorea* was higher among American patients with MS, which was inconsistent with the findings of the present study (9). The anti-inflammatory properties of *Dorea* might be associated with the development of MS. *Butyricicoccus*, which is also a butyrate producer, is reportedly a beneficial bacterium that suppresses inflammatory bowel diseases, which has a strong negative correlation with the levels of the target cytokines and cell types (78). Shi et al. observed that the abundance of *Butyricicoccus* decreased significantly in patients with severe and active Graves’ orbitopathy compared to that in controls (79). Everard et al. demonstrated that the abundance of *Butyricicoccus*, which can improve gut barrier integrity, correlated negatively with the levels of pathogenic Aβ42 in the brain (80). Therefore, our present study revealed that altered composition of the fecal microbiota especially those SCFAs producers, which regulate host immune homeostasis, might play vital roles in the development of MS.

In fact, systemic inflammation can also affect disease expression in MS. The progression of MS is associated with the loss of immune homeostasis, increased systemic inflammation, and disruption of the blood-brain barrier. A previous study has revealed that CD4^+^ T cells mediate inflammation in the central nervous system, which triggers demyelination and axonal degeneration, and this has been shown to play a major role in MS pathogenesis (81). Consistent with previous studies, our present study also revealed obvious systemic inflammation in Chinese patients with MS, characterized by increased levels of IL-17 and TNF-α and decreased levels of IL-1ra, IL-8, IL-9, MCP-1, MIP-1a, and MIP-1b. The changes in the levels of inflammatory mediators such as inflammatory and anti-inflammatory cytokines, chemokines, and growth factors might constitute the primary characteristics of systemic inflammation in MS patients, and current MS therapies aim to restore host immunity to a healthy status. Kürtüncü et al. observed that increased IL-17 levels can be reduced significantly after IFN-β treatment, which indicates that Th17-type immunity plays a crucial role in the pathogenesis of MS. Recently, the chemokine IL-9 has been shown to play a major role in regulating autoimmune responses in experimental autoimmune encephalomyelitis, which is an animal model of MS. IL-9 has emerged as a key cytokine involved in the regulation of the balance between Th17 and Tregs levels in MS (82). The intestinal microbiota can influence host immunity in the intestinal tract as well as at distal sites. The intestinal microbiota affect systemic immune responses by modulating several key pathways: expansion of extra-intestinal T cell populations, production of SCFAs, development of oral tolerance, and control of inflammation (83). In our study, we found that the abundance of *Faecalibacterium* correlated positively with the levels of IL-8 and MIP-1a. IL-8 possesses potent neutrophil chemoattractant and activating properties. A previous study showed that butyrate, mainly metabolites of *Faecalibacterium*, can induce Caco-2 cells to secrete IL-8 in response to lipopolysaccharide (84). However, recent study found that the supernatant of *Faecalibacterium* culture can NF-κB activation and the production of the pro-inflammatory cytokine IL-8 *in vitro*, which is contrary to our present findings (85). The abundances of *Faecalibacterium* and *Roseburia* correlated negatively with the levels of the pro-inflammatory cytokine TNF-α, while that of *Flavonifractor*, which is an MS-enriched taxa, correlated positively with the levels of TNF-α. TNF-α can mediate monocyte infiltration into the intestinal tissues resulting in tissue damage, which is one signs of inflammation. Interestingly, *Faecalibacterium* and its supernatant can suppress the expression TNF-α, and then alleviate the inflammation. Evidence has shown that *Roseburia* plays an important role in maintaining gut health by improving the gut ecosystem and exhibiting anti-inflammatory effects (86, 87). The correlations between *Roseburia* and TNF-α in our present findings might also link with its metabolite such as butyrate. *Flavonifractor* has been reported to participate in the metabolism of catechin in the gut (88). However, the biological regulatory effects of *Flavonifractor* in autoimmune diseases are still unclear. We postulated that *Flavonifractor* might influence the expression of TNF-α directly or *via* its metabolites. Of course, the systemic immune response in MS was impacted by the bacterial mixtures of these key functional bacteria together. The specific roles of these key functional bacteria alone should be explored in MS animal models in the future. Interestingly, numerous studies have demonstrated that the metabolites of these key functional bacteria, SCFAs, have been demonstrated to influence systemic autoimmune responses and participate in different steps of the inflammatory process (89, 90). SCFAs have been observed to regulate the functions of almost every type of immune cell by altering gene expression, differentiation, chemotaxis, proliferation, and apoptosis. Previous studies have shown that compared to acetate and propionate, butyrate exhibits strong anti-inflammatory properties, which exert regulatory effects on inflammatory processes by maintaining the balance of Th17/Treg cells and the levels of pro- and anti-inflammatory cytokines (91). These data indicate that the disturbed intestinal microbiota, especially the SCFA-producing key intestinal functional bacteria that are variably abundant, could modulate host immunity, induce systemic inflammation, and participate in the progression of MS. Therefore, this is a useful strategy that targets systemic inflammation by modulating the abundances of specific intestinal key functional bacteria, and may provide novel avenues for MS-modifying therapy.

However, our present study is limited in some ways. First, the number of MS patients was relatively small in our present microbiota analysis. More MS patients enrolled from different regions of China might make our results more solid and reasonable. Second, our 16S rRNA amplicon rather than metagenomic sequencing limited the finding of specific bacteria related to MS at the species level. Third, the functional and metabolic changes in the microbial communities were mostly speculative and there was no functional validation of the microbiome findings. Future culturomics should be used to obtain the MS-associated bacteria, and cell and animal experiments could help determine the cause-effect relationship between these key functional bacteria and the pathogenesis of MS.

## Conclusion

In summary, our study revealed alterations in the fecal microbiota and altered host immune response in Chinese patients with stable MS. The characterization and manipulation of the MS-associated microbiome may have significant potential as diagnostic and therapeutic strategies, respectively. In addition, the abundances of key functional bacteria that were altered significantly correlated with the levels of pro- and anti-inflammatory cytokines and chemokines, which indicates that intestinal microbiota might play vital roles in the development of MS *via* the regulation of host immunity. Therefore, the modulation of intestinal microbiota through personalized diet or beneficial microbial intervention may be a potential strategy for patient-tailored early intervention of MS. However, the role of those key functional bacteria especially butyrate producers in systemic inflammation in Chinese patients with MS in the present study remains unclear. Further studies are necessary to decode the sophisticated bidirectional dialogue between butyrate-producing bacteria and host immunity associated with the development, remission, and prognosis of Chinese patients with MS.

## Data Availability Statement

The datasets presented in this study can be found in online repositories. The names of the repository/repositories and accession number(s) can be found in the article/**Supplementary Material**.

## Ethics Statement

The studies involving human participants were reviewed and approved by the Ethics Committee of Lishui Second People's Hospital (Zhejiang, China). The patients/participants provided their written informed consent to participate in this study.

## Author Contributions

ZL, XY, and LYZ conceived and designed the experiments. ZL, XY, YC, SL, XL, DZ, LJZ, and KQ performed the experiments. ZL, XY, LS, and XL analyzed the data. ZL, YC, and LYZ wrote the paper and edited the manuscript. All authors contributed to the article and approved the submitted version.

## Funding

This present work was funded by the grants of the National S&T Major Project of China (2018YFC2000500), the National Natural Science Foundation of China (81771724, 31700800, 81790631), the S&T Major Project of Lishui (2017ZDYF15), and the Foundation of China’s State Key Laboratory for Diagnosis and Treatment of Infectious Diseases. The authors thank all of the participants who recruited patients in this study.

## Conflict of Interest

The authors declare that the research was conducted in the absence of any commercial or financial relationships that could be construed as a potential conflict of interest.

## Supplementary Material

Supplementary Figure 1 The diversity and richness of the fecal microbiota in MS. The diversity indices, such as Shannon **(A)**, and Simpson **(B)**, and the richness indices, such as observed species **(C)** ACE **(D)**, and Chao1 **(E)**, were used to evaluate the overall structure of the fecal microbiota in Chinese MS patients and healthy controls. Data are presented as mean ± standard deviation. Unpaired *t* tests (two-tailed) were used to analyze variation between the two groups. Principal coordinate analysis (PCoA) plots of individual fecal microbiota based on jaccard **(F)**, Bray-Curtis **(G)**, unweighted **(H)** and weighted (**I**) UniFrac distance in Chinese MS patients and healthy controls. Each symbol represents a sample. The Venn diagram illustrates the overlap of OTUs in the fecal microbiota between the two groups **(J)**. Rarefaction curves were used to estimate the richness (at a 97% level of similarity) of the fecal microbiota between the two groups **(K)**. The vertical axis shows the number of OTUs expected after sampling the number of tags or sequences shown on the horizontal axis. Rank abundance curves of bacterial OTUs derived from the two groups, which indicated that the majority of the OTUs were present at low abundance in the fecal microbiota samples with greater sequencing depth **(L)**.

Supplementary Figure 2 Heatmap of the family-level taxa in the fecal microbiota of Chinese MS patients and healthy controls. The colour of the spots in the panel represents the relative abundance (normalized and log10-transformed) of the family in each sample. The relative abundance of the bacteria in each family is indicated by a gradient of colour from blue (low abundance) to red (high abundance). The taxonomic classifications of the family are shown on the right. The corresponding Shannon index in each sample is shown under the heatmap.
