# Supplementary figures and images for: Alterations of the Fecal Microbiota in Chinese Patients With Multiple Sclerosis
Source: Front Immunol. 2020 Dec 16;11:590783. doi: 10.3389/fimmu.2020.590783 (PMC7772405; doi:10.3389/fimmu.2020.590783)

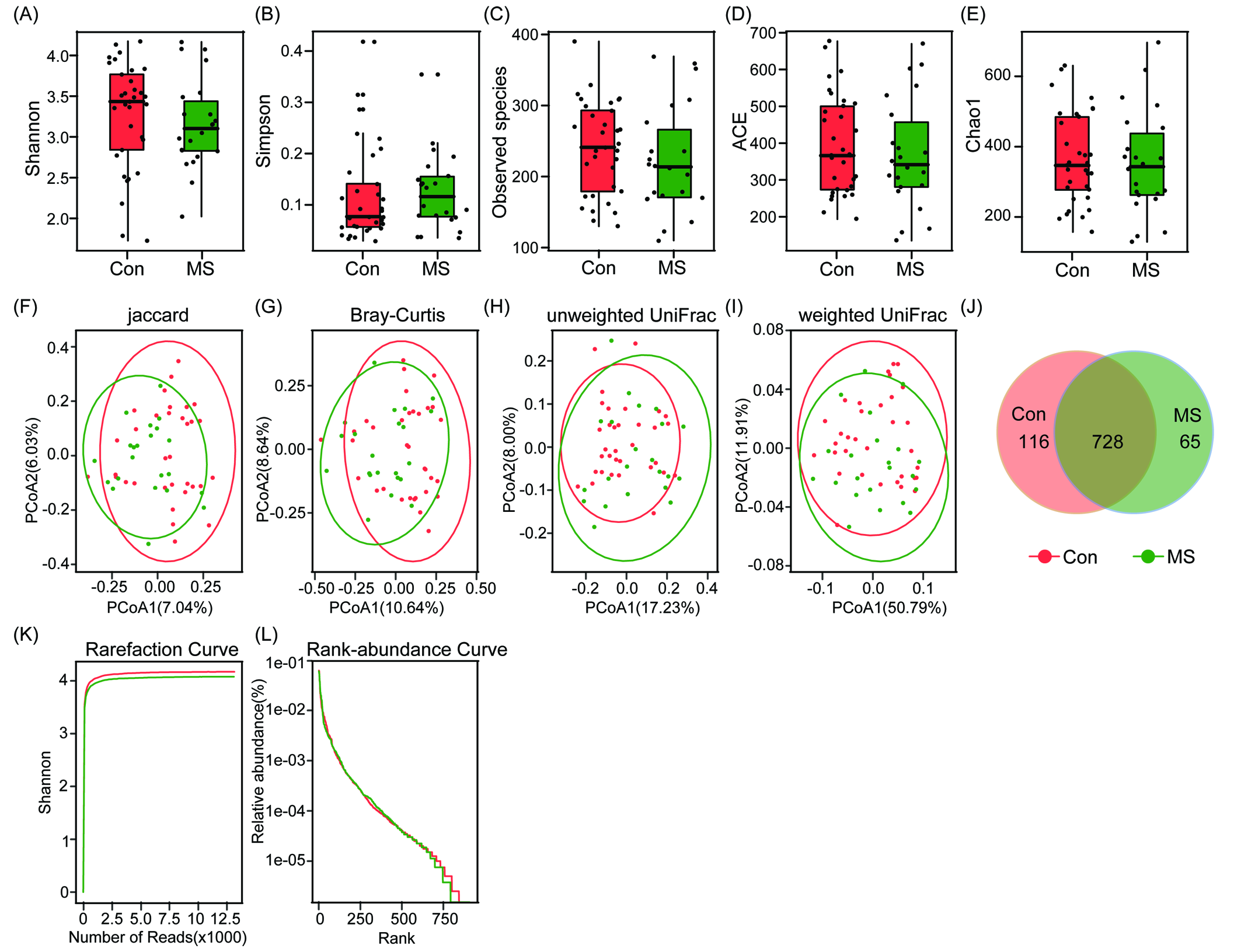

Supplement: Supplementary Figure 1 — The diversity and richness of the fecal microbiota in MS. The diversity indices, such as Shannon (A), and Simpson (B), and the richness indices, such as observed species (C) ACE (D), and Chao1 (E), were used to evaluate the overall structure of the fecal microbiota in Chinese MS patients and healthy controls. Data are presented as mean ± standard deviation. Unpaired t tests (two-tailed) were used to analyze variation between the two groups. Principal coordinate analysis (PCoA) plots of individual fecal microbiota based on jaccard (F), Bray-Curtis (G), unweighted (H) and weighted (I) UniFrac distance in Chinese MS patients and healthy controls. Each symbol represents a sample. The Venn diagram illustrates the overlap of OTUs in the fecal microbiota between the two groups (J). Rarefaction curves were used to estimate the richness (at a 97% level of similarity) of the fecal microbiota between the two groups (K). The vertical axis shows the number of OTUs expected after sampling the number of tags or sequences shown on the horizontal axis. Rank abundance curves of bacterial OTUs derived from the two groups, which indicated that the majority of the OTUs were present at low abundance in the fecal microbiota samples with greater sequencing depth (L). [file Image_1.tif]

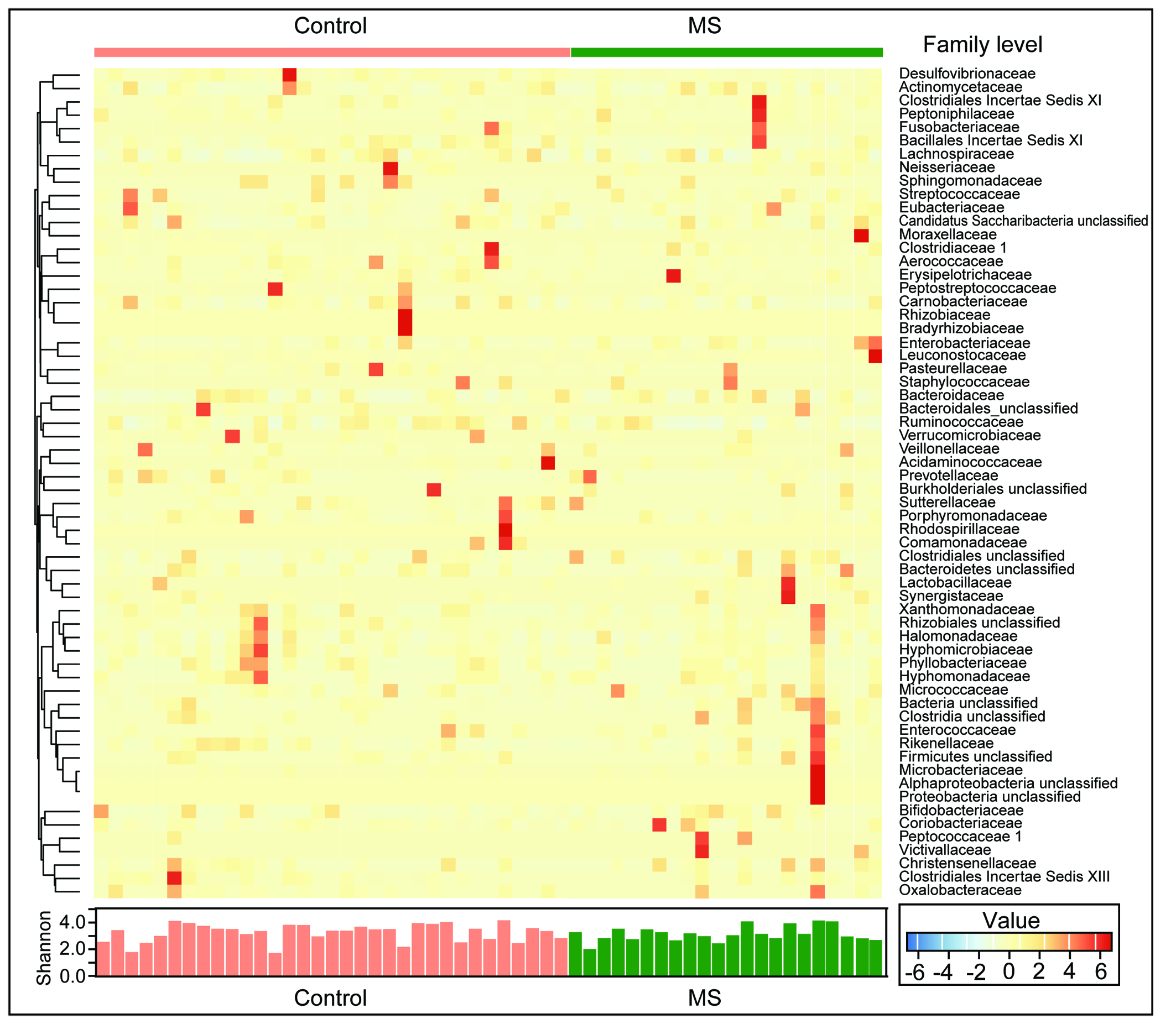

Supplement: Supplementary Figure 2 — Heatmap of the family-level taxa in the fecal microbiota of Chinese MS patients and healthy controls. The colour of the spots in the panel represents the relative abundance (normalized and log10-transformed) of the family in each sample. The relative abundance of the bacteria in each family is indicated by a gradient of colour from blue (low abundance) to red (high abundance). The taxonomic classifications of the family are shown on the right. The corresponding Shannon index in each sample is shown under the heatmap. [file Image_2.tif]
